# Predictive value of neutrophil to lymphocyte ratio and red cell distribution width on death for ST segment elevation myocardial infarction

**DOI:** 10.1038/s41598-021-91082-w

**Published:** 2021-06-01

**Authors:** Guoli Lin, Caizhi Dai, Kaizu Xu, Meifang Wu

**Affiliations:** Department of Cardiology, The Affiliated Hospital of Putian University, Putian University, No. 999 Dongzhen East Road, Licheng District, Putian, 351100 Fujian China

**Keywords:** Interventional cardiology, Predictive markers

## Abstract

There are many clinical scoring criteria for predicting the risk of death in patients with acute ST-segment elevation myocardial infarction (STEMI), but most of the indicators are complex to calculate and are not suitable for use in primary hospitals. Neutrophil to lymphocyte ratio (NLR) and red cell distribution width (RDW) are blood routine indicators that are easy to obtain and may help primary hospitals to evaluate the risk of death in patients with STEMI. Our aim was to explore the predictive value of NLR combined with RDW in the long-term prognosis of patients with STEMI after emergency percutaneous coronary intervention (PCI). A total of 181 patients with STEMI who underwent emergency PCI in the Affiliated Hospital of Pu-tian University from January 2017 to August 2018 were selected. Clinical profile, prognosis of all patients were collected. *P* value < 0.05 was considered significant. In all patients, cardiovascular death during the follow-up period was defined as cardiovascular death group, and surviving during the follow-up period was defined as survival group. There were no significant differences in demography and comorbidities between the two groups. The differences between the two groups in NLR, RDW, C-reactive protein, N-terminal-pro B type natriuretic peptide were statistically significant (*P* < 0.01). Binary logistic regression analysis showed that NLR (OR = 1.122, 95% CI 1.041 ~ 1.210, *P* = 0.003) and RDW (OR = 1.288, 95% CI 1.126 ~ 1.472, *P* = 0.0005) were important predictors of mortality in patients with STEMI (*P* < 0.05). Kaplan–Meier analysis showed that as the NLR increased, the risk of death increased (*P* < 0.001). In conclusion, NLR and RDW are independent predictors of cardiovascular death in patients with STEMI, and they have a certain predictive value.

## Introduction

Acute ST segment elevation myocardial infarction (STEMI) is a serious type of coronary heart disease and the main cause of death and disability. The incidence of STEMI in China is showing a rapid increase. From 2001 to 2011, the hospitalization rate of STEMI patients in China increased nearly fourfold (from 4.6/100,000 to 18/100,000 for males and from 1.9/100,000 to 8/100,000 for females). Since 2013, the mortality rate of acute myocardial infarction in rural areas has been significantly higher than that in urban areas^1^. In the "China Cardiovascular Health and Disease Report 2019", it is estimated that the number of people with cardiovascular disease in China is 330 million, and nearly 700,000 cases of acute myocardial infarction occur every year.

STEMI refers to acute myocardial ischemic necrosis, which can lead to acute inflammation and stress response characterized by increased leukocyte mobilization in the area of myocardial necrosis^2^. White blood cell count within 24 h after admission is an independent predictor of death and major adverse events in patients with acute myocardial infarction^3^, and low levels of lymphocytes are also significantly associated with cardiovascular disease mortality^4^. The neutrophil-to-lymphocyte ratio (NLR) is an indicator of inflammation that is more valuable than a single indicator in predicting the prognosis of patients with AMI. In non-STEMI patients, NLR is a very valuable predictor of hospital mortality^5^. The Oh PC study suggests that NLR can be used to indicate the severity of myocardial injury and whether there is adverse cardiac remodeling. In STEMI patients, high NLR is independently associated with the risk of death at 1 year after PCI^6^. Related studies have shown that NLR can not only predict the long-term mortality of patients with acute ST-segment elevation myocardial infarction^7^, but also reflect the severity of coronary artery disease, and is related to the SYNTAX score and Gensini score^8^. A study also proved that NLR is an independent predictor of no reflow and an indicator of hospitalization and long-term prognosis^9^. In STEMI patients, NLR is an independent predictor of high residual SYNTAX score and an independent predictor of the severity of coronary artery disease after PCI^10^. Among the 60,087 participants in five contemporary randomized trials, NLR consistently predicted future cardiovascular events and all-cause mortality^11^.

Red cell distribution width (RDW) represents the coefficient of variation of the width of the erythrocyte volume distribution and is a measure of erythrocyte size variability. There is evidence that RDW is a new biomarker of inflammation and oxidative stress in the circulation, and has become a risk predictor of various cardiovascular diseases. RDW has been proven to be an important independent predictor of cardiovascular events in patients with coronary heart disease, including ACS, and also a predictor of cardiovascular death and all-cause death^12^. Polat N’s research shows that high RDW is an independent predictor of high GRACE scores and is associated with Unstable angina pectoris/non-STEMI in-hospital mortality. There was a significant correlation between RDW and GRACE risk group in terms of mortality from hospitalization and discharge to 6 months^13^.

At present, there are few reports on the relationship between NLR and RDW on the long-term prognosis of patients with acute ST segment elevation myocardial infarction. Based on the analysis of the results of 2-year follow-up of STEMI patients with percutaneous coronary intervention (PCI), this study discussed the predictive value of NLR and RDW in the long-term prognosis of these patients, in order to establish a simple, non-invasive, cost-effective method to evaluate the prognosis and inform clinical decision making.

## Methods

### Study populations

This study was in accordance with the Declaration of Helsinki and was approved by the hospital’s ethical review board (The Affiliated Hospital of Putian University, Putian University, Fujian, China).

This is a retrospective research, the data for patients were extracted from the hospital digital information system. These data did not involve identifiable personal data such as the patient’s name but did provide the age, sex, number of patients, and the national classification code of disease. Therefore, the approved our study without the need to obtain informed consent from patients.

One hundred and eighty-one patients with acute ST segment elevation myocardial infarction who underwent emergency PCI in The Affiliated Hospital of Putian University from January 2017 to August 2018 were selected. The diagnosis of acute ST segment elevation myocardial infarction is in accordance with the guidelines for diagnosis and treatment of acute ST segment elevation myocardial infarction published by Chinese Medical Association in 2015^14^. Exclusion criteria: accompanied by active infection, chronic inflammatory diseases, liver and kidney failure, malignant tumors, hematological diseases, autoimmune diseases. All patients were followed up for 2 years. Cardiovascular death during the follow-up period was defined as cardiovascular death group, and surviving during the follow-up period is defined as survival group.

### Data collection

All data were obtained from the hospital digital information system. Demographic and clinical parameters, laboratory test results and PCI data were collected. All data were obtained from medical records and PCI records. Automatic cell analyzer was used for Hematologic (Mindray CAL8000, Mindray, Shenzhen, China), and automatic biochemical analyzer (GeteinBiotech CM-800, GeteinBiotech, Nanjing, China) was used for biochemical indices. The values of neutrophils and lymphocytes in the blood routine at the time of admission were selected to calculate the NLR value. The angiographic results were evaluated by quantitative coronary artery measurement, and the PCI operation method was determined by experienced operators. The above information comes from the interventional catheterization laboratory and information center of The Affiliated Hospital of Pu-tian University.

### Follow-up

After PCI, the patients were followed up at hospitalization, discharge for 1 month, 6 months, 1 year, 1.5 years and 2 years, respectively. The methods of follow-up included outpatient follow-up, telephone follow-up, recording of Cardiovascular death event and termination of follow-up after the occurrence of Cardiovascular death event. Patients who did die from other causes rather than cardiovascular disease were excluded.

### Statistical analysis

All statistical analyses were conducted using Statistical Package for Social Sciences software (SPSS 18.0 for Windows, IBM, USA). Continuous variables are presented as mean ± standard deviation and are compared using independent-samples t test. Categorical variables were expressed as proportions and the differences in categorical variables were analyzed using chi-square test. Logistic regression analysis was used to analyze the risk factors of cardiovascular death event. Receiver operating characteristics (ROC) curve analysis and area under the curve (AUC) calculations of NLR and RDW were used to calculate their effectiveness in predicting mortality. Survival analysis was conducted using Kaplan–Meier survival curves, and differences were compared using the log-rank test. *P* value < 0.05 was considered statistically significant.

## Results

### Baseline characteristics of patients

There were 21 males and 3 females in the cardiovascular death group, with an average age of 68.19 ± 10.72 years old, including 7 cases with hypertension and 5 cases with diabetes. In the survival group, there were 131 males and 26 females with an average age of 63.57 ± 11.63 years old. There were 53 cases of hypertension and 34 cases of diabetes mellitus. There was no statistically significant difference between the two groups in age, gender, comorbidities, and lesion vessel (*P* > 0.05). There were no significant differences in heart rate, diastolic blood pressure, days of hospitalization, and the number of implanted stents at admission. The systolic blood pressure of the cardiovascular death group was higher than that of the survival group. The values were shown in Table 1.

**Table 1:** Clinical and demographic properties of two groups.

Variable	Cardiovascular death group (n = 24)	Survival group (n = 157)	*P* value
Age (years)	68.19 ± 10.72	63.57 ± 11.63	0.063
Male (%)	21(87.50)	131(83.44)	0.771
Hypertension (%)	7(29.17)	53(33.76)	0.817
Diabetes (%)	5(20.83)	34(21.66)	0.584
LM (%)	1(0.04)	3(0.02)	0.437
LAD (%)	13(0.54)	76(0.48)	0.664
LCX (%)	0(0)	17(0.10)	0.134
RCA (%)	10(0.42)	60(0.38)	0.823
Heart rate (beats/min)	75.91 ± 16.71	82.50 ± 21.19	0.085
SBP (mmHg)	125.90 ± 24.98	112.88 ± 30.63	0.022
DBP (mmHg)	77.41 ± 16.23	72.63 ± 17.70	0.185
Hospitalization days	9.71 ± 4.11	10.13 ± 7.26	0.681
Number of stents	1.43 ± 0.69	1.63 ± 0.88	0.209

DBP, diastolic blood pressure; LAD, left anterior descending artery; LCX, left circumflex artery; LM, left main coronary artery; RCA, right coronary artery; SBP, systolic blood pressure.

### Laboratory parameters of patients

There were significant differences in NLR, white blood cell, erythrocyte, hemoglobin, RDW, blood glucose, creatinine, D-dimer, fibrin degradation products, CRP and Nt-pro-BNP between the two groups. There were no significant difference in mean red blood cell volume, total platelet count, platelet volume distribution width (PDW), lymphocyte counts, total bilirubin, triglyceride, total cholesterol, high-density lipoprotein cholesterol, low-density lipoprotein cholesterol, uric acid, troponin, glycosylated hemoglobin and thyroid stimulating hormone between the two groups. The values were shown in Table 2.

**Table 2:** Comparison of two groups of laboratory parameters.

Variable	Cardiovascular death group(n = 24)	Survival group(n = 157)	*P* value
NLR	10.44 ± 4.85	7.07 ± 5.24	0.003
WBC count (10^9^/L)	16.69 ± 10.36	11.21 ± 3.96	0.017
RBC count (10^9^/L)	4.28 ± 0.62	4.61 ± 0.62	0.016
Hemoglobin (g/L)	128.68 ± 19.78	138.73 ± 17.84	0.012
MCV (fl)	89.73 ± 8.68	88.73 ± 5.51	0.448
RDW (%)	13.56 ± 1.54	12.73 ± 0.98	0.001
Platelet count (10^9^/L)	233.20 ± 76.34	241.16 ± 82.82	0.641
PDW (%)	11.00 ± 2.93	11.46 ± 2.29	0.382
Lymphocyte counts (10^9^/L)	1.53 ± 1.39	1.67 ± 0.90	0.525
TB (umol/L)	10.53 ± 5.51	10.18 ± 4.32	0.724
TG (mmol/L)	1.21 ± 0.80	1.81 ± 2.42	0.230
TC (mmol/L)	4.42 ± 1.34	4.80 ± 1.37	0.196
HDL (mmol/L)	1.02 ± 0.33	1.05 ± 0.27	0.674
LDL (mmol/L)	3.12 ± 1.10	3.026 ± 1.14	0.567
Glucose (mmol/L)	10.17 ± 4.39	8.25 ± 3.28	0.012
Creatinine (umol/L)	85.55 ± 31.33	75.13 ± 22.70	0.049
Uric acid (umol/L)	380.58 ± 134.81	373.49 ± 98.82	0.757
D-dimer (ug/mL)	3.69 ± 8.16	0.55 ± 1.06	0.001
FDP (ug/mL)	12.53 ± 34.35	1.60 ± 2.26	0.001
CRP (μg/L)	26.71 ± 40.56	10.53 ± 20.87	0.03
*Peak* Tn-I (ng/ml)	34.93 ± 36.03	34.30 ± 33.09	0.932
Nt-pro-bnp (Pg/ml)	2680.29 ± 4656.15	1053.55 ± 2887.96	0.021
TSH (uIU/ml)	0.87 ± 0.46	1.28 ± 1.37	0.248
HbA_1_c (%)	7.02 ± 1.89	7.20 ± 1.95	0.717
*Peak* CK-MB(U/L)	223.79 ± 64.37	194.33 ± 70.37	0.055
*Peak* CK(U/L)	2067.17 ± 1744.96	1352.97 ± 1649.45	0.070

CK, Creatine Kinase; CK-MB, Creatine Kinase Isoenzyme-MB; CRP, C-reactive protein; FDP, fibrin degradation products; HbA_1_c, hemoglobin A_1_c; HDL-C, high-density lipoprotein cholesterol; LDL-C, low-density lipoprotein cholesterol; MCV, mean red blood cell volume; NLR, neutrophil to lymphocyte ratio; Nt-pro-BNP, N terminal pro B type natriuretic peptide; PDW, platelet volume distribution width; RBC, red blood cell; RDW, red cell distribution width; TB, total bilirubin; TC, total cholesterol; TG, triglyceride; Tn-I, Troponin I; TSH, thyroid stimulating hormone; WBC, white blood cell.

### NLR and RDW are independent predictors of cardiovascular death

Binary logistic regression analysis showed that NLR (OR = 1.122, 95% CI 1.041 ~ 1.210, *P* = 0.003) and RDW (OR = 1.288, 95% CI 1.126 ~ 1.472, *P* = 0.0005) were important predictors of mortality in patients with STEMI (*P* < 0.05). The values were shown in Table 3.

**Table 3:** Logistic regression analysis for cardiovascular death.

Variables	β	Wals	*P*	OR	95% CI
NLR	0.115	9.018	0.003	1.122	1.041 ~ 1.210
RDW	0.253	13.694	0.0005	1.288	1.126 ~ 1.472
Peak Tn-I	0.013	3.882	0.049	1.013	1.000 ~ 1.025
Peak CK-MB	0.011	4.099	0.043	1.011	1.000 ~ 1.022
Peak CK	0.001	4.337	0.037	1.001	1.000 ~ 1.002

CK, Creatine Kinase; CK-MB, Creatine Kinase Isoenzyme-MB; NLR, neutrophil to lymphocyte ratio; RDW, red cell distribution width; Tn-I, Troponin I.

### Receiver operating characteristic curve

**Figure 1:**
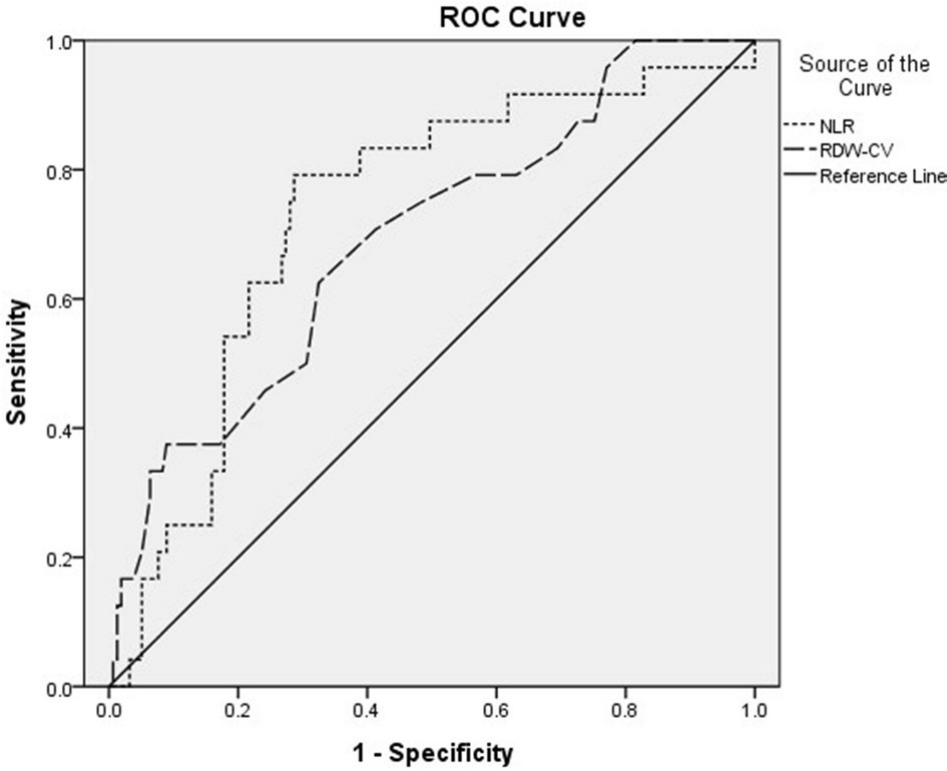
Receiver operating curve showing the AUC for NLR and RDW. NLR, neutrophil to lymphocyte ratio; RDW, red cell distribution width.

Area under the curve of NLR was 0.732 (95% CI: 0.626 ~ 0.838, *P* < 0.001), the best cut-point value for predicting cardiovascular death was 8.16, sensitivity was 79%, and specificity was 72%; Area under the curve of RDW was 0.692 (95% CI: 0.578 ~ 0.806, *P* < 0.001), the best cut-point value for predicting cardiovascular death was 12.75, sensitivity was 71%, and specificity was 60% (Fig. 1).

### The Kaplan–Meier survival analysis for cardiovascular death

**Figure 2:**
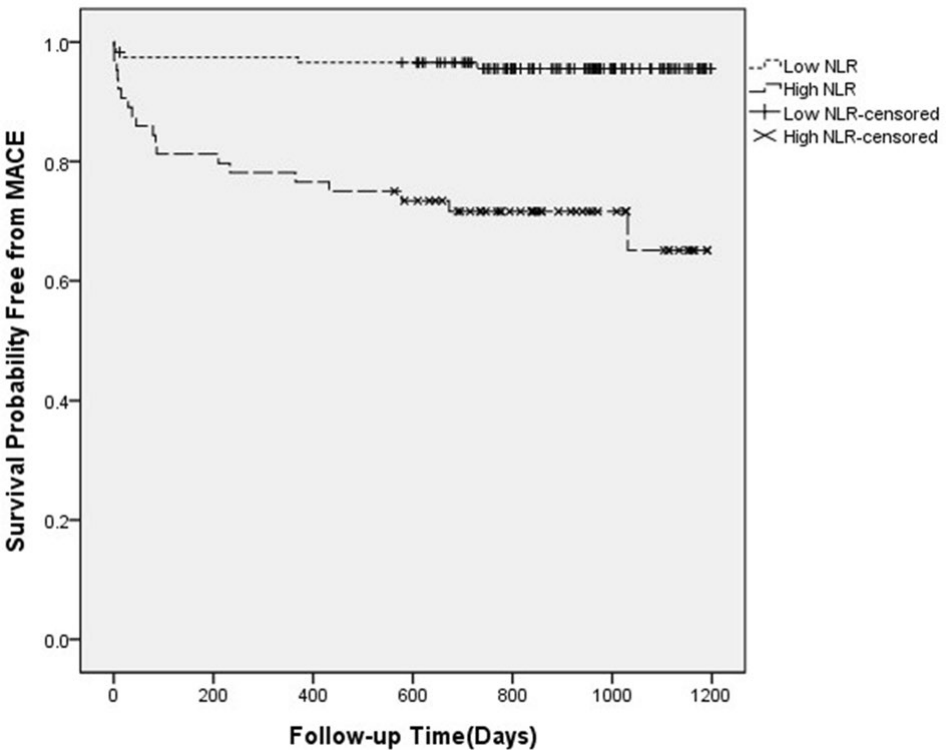
Kaplan–Meier survival curve of 2-year cardiovascular death in patients with high NLR and low NLR groups. NLR, neutrophil to lymphocyte ratio.

ROC curve analysis showed that the best critical value of NLR for predicting 2-year mortality was 8.16. According to this, the patients were divided into high NLR (NLR ≥ 8.16) group (n = 64) and low NLR (NLR < 8.16) group (n = 117). The results of 2-year follow-up showed that the incidence of cardiac mortality in the high NLR group was higher than that in the low NLR group (*P* < 0.001) (Table 4). The Kaplan survival Meier analysis showed the same trend (Fig. 2).

**Table 4:** Two-year follow-up results of patients in the high and low NLR groups.

	Cardiovascular death group	Survival group
High NLR group (n = 64)	19(29.68)	45 (70.32)
Low NLR group (n = 117)	5(0.04)	112 (0.96)
X^2^	–	23.23
*P* value	–	< 0.001

NLR, neutrophil to lymphocyte ratio.

## Discussion

With economic development and changes in lifestyle, acute ST-segment elevation myocardial infarction has a high morbidity, disability and mortality rate in China. At present, there is still a lack of easy-to-obtain biomarkers to quickly identify high-risk populations of death from acute myocardial infarction. This biomarker is essential for screening, intensive treatment and reducing the occurrence of adverse events in high-risk groups.

Early diagnosis can provide better guidance for patients with acute myocardial infarction. It has been reported that lymphocytes and monocytes in STEMI patients undergoing PCI are independent predictors of major in-hospital and long-term adverse cardiovascular and cerebrovascular events^15^. White blood cell count, C-reactive protein and neutrophil percentage can predict the mortality of patients with ACS at the same time. The increase of neutrophils after acute myocardial infarction may be related to the occurrence of congestive heart failure^16^. The ratio of neutrophils to lymphocytes is one of the indicators of inflammation, and it has been proven that this factor can be used in many diseases. In cardiovascular disease, NLR is an independent predictor of ventricular dysfunction and is associated with the severity and mortality of coronary heart disease^17^. Acet H's study showed that higher NLR levels were significantly related to the decreased patency of infarct-related blood vessels before PCI, suggesting that NLR may be a better indicator of the presence of infarct-related artery blood flow in STEMI patients before PCI^18^. High NLR increased significantly with the increase of GRACE risk score, suggesting that NLR may be an indicator of GRACE risk score in STEMI patients. The GRACE score has been considered to be an effective predictor of adverse cardiovascular events in patients with cardiovascular diseases^19^. The study found that in STEMI patients, NLR was positively correlated with TIMI score, and TIMI score was strongly correlated with mortality within 30 days^20^. In elderly patients with AMI, NLR is significantly and independently related to mortality, and has better predictive power than other leukocyte parameters. Moreover, adding NLR to traditional risk models, including age, hypertension, diabetes and other risk factors, also significantly improves the efficiency of the model^21^. Recent studies have found that in acute coronary syndromes, the NLR value is related to the prognosis of ACS. The higher the NLR value, the worse the prognosis of ACS patients^22^. In non-STEMI patients, elevated NLR before PCI is associated with a high risk of clinical adverse events within 1 year^23^. The possible mechanisms are as follows: (1) NLR is associated with increased inflammatory activity, and neutrophils secrete a large number of inflammatory mediators, which participate in the acute inflammatory response in the site of tissue injury^24^. In the process of occurrence and development of acute myocardial infarction, the body is in a state of inflammatory activation, the level of blood cortisol increases, and the increased cortisol induces apoptosis, resulting in lymphopenia and CD^4+^/CD^8+^ T lymphocyte ratio inversion. Lymphocytopenia in CD^4+^ is clearly related to poor prognosis in STEMI^25^. Neutrophils and monocytes play a key role in the inflammatory response, while lymphocytes are involved in the regulation of the immune system. NLR may be more predictive of the occurrence of diseases than a single white blood cell count, and has more clinical predictive value. We found that the NLR value in the cardiovascular death group was significantly higher than that in the survival group. Logistic regression analysis showed that NLR was a risk factor for predicting cardiovascular death events, and the high NLR group had a higher risk of cardiovascular death, which was consistent with the above point of view.

RDW represents the coefficient of variation of red blood cell volume distribution width. The mechanisms that affect RDW levels include inflammatory stress, adrenergic activation, nutritional deficiency and iron homeostasis disorder. Many studies have found that increased RDW is a powerful independent predictor of cardiovascular events in heart disease patients including ACS. The increase of RDW indicates a decrease in erythrocyte deformability and damage of microcirculatory blood flow, resulting in a decrease in oxygen supply at the tissue level, which may be related to an increase in the risk of cardiovascular adverse events. An increase in the level of RDW can increase the morbidity and all-cause mortality of cardiovascular disease^26^. An increase in RDW has been reported to predict not only the poor outcome of thrombolytic therapy for STEMI, but also the long-term poor prognosis of STEMI patients treated with PCI^27^. RDW is positively correlated with Gensini score, it is significantly correlated with the severity of ACS, and it is an independent predictor of MACE in ACS patients^28^. The inflammatory process and subsequent release of cytokines in acute myocardial infarction increase oxidative stress, reduce the lifespan of red blood cells, make immature red blood cells enter the blood from bone marrow, damage the mature erythrocyte membrane and change ion channel glycoproteins, thus affecting the level of RDW. Our study showed that the RDW value of the cardiovascular death group was significantly higher than that of the survival group. Logistic regression analysis indicated that RDW is also a risk factor for predicting cardiovascular death events, which is consistent with the above view.

In this study, the levels of NLR and RDW in cardiovascular death group were higher than those in survival group. According to logistics regression analysis, NLR and RDW are considered to be independent predictors of cardiovascular death. Kaplan–Meier analysis shows that NLR can be used as an important predictor of long-term poor prognosis. The predictive value of NLR and RDW for cardiovascular mortality was shown by ROC curves. The results showed that the area under the curve for NLR was 0.732, the best cut point value for predicting cardiovascular death was 8.16, with a sensitivity of 79% and specificity of 72%; the area under the curve for RDW was 0.692, the best cut point for RDW for predicting cardiovascular death was 12.75, with a sensitivity of 71% and specificity of 60%. It has a certain diagnostic value for long-term death of STEMI patients.

Our study showed that the best cut point value for NLR to predict cardiovascular death was 8.16. The value differs from other literature and may be related to each hospital's testing method and equipment. Therefore, different hospitals need to calculate NLR values according to their own conditions to predict cardiovascular death.

## Conclusions

NLR and RDW have a certain predictive value in evaluating the occurrence of cardiovascular death after PCI in patients with acute ST segment elevation myocardial infarction. This method has the advantages of convenient detection and low cost, and has a good application prospect in predicting cardiovascular death after PCI in patients with STEMI.

There are some limitations in this study. First of all, this study only selected NLR and RDW in the blood routine at admission as the observation indicators and lack of comparison, it is best to measure multiple times and conduct comparative studies. Secondly, the short 2-year follow-up time and the small number of cases in this study can also affect the results. Finally, this study is not a randomized controlled study, and a large-scale randomized controlled study is still needed to evaluate the predictive value of NLR and RDW on the death of STEMI patients.

## Data Availability

All data generated or analysed during this study are included in this published article.
